# Elevated Black Carbon Concentrations and Atmospheric Pollution around Singrauli Coal-Fired Thermal Power Plants (India) Using Ground and Satellite Data

**DOI:** 10.3390/ijerph15112472

**Published:** 2018-11-05

**Authors:** Ramesh P. Singh, Sarvan Kumar, Abhay K. Singh

**Affiliations:** 1 School of Life and Environmental Sciences, Schmid College of Science and Technology, Chapman University, Orange, CA 92866, USA; 2 Indian Institute of Tropical Meteorology, Dr. Homi Bhabha Road, Pashan, Pune 411008, India; sarvanbhaskar07@gmail.com; 3 Atmospheric Research Lab., Department of Physics, BHU, Varanasi 221005, India; abhay_s@rediffmail.com

**Keywords:** black carbon, AOD, power plants, coal mining, CALIPSO, Indo-Gangetic Plain

## Abstract

The tropospheric NO_2_ concentration from OMI AURA always shows high concentrations of NO_2_ at a few locations in India, one of the high concentrations of NO_2_ hotspots is associated with the locations of seven coal-fired Thermal Power plants (TPPs) in Singrauli. Emissions from TPPs are among the major sources of black carbon (BC) soot in the atmosphere. Knowledge of BC emissions from TPPs is important in characterizing regional carbonaceous particulate emissions, understanding the fog/haze/smog formation, evaluating regional climate forcing, modeling aerosol optical parameters and concentrations of black carbon, and evaluating human health. Furthermore, elevated BC concentrations, over the Indo-Gangetic Plain (IGP) and the Himalayan foothills, have emerged as an important subject to estimate the effects of deposition and atmospheric warming of BC on the accelerated melting of snow and glaciers in the Himalaya. For the first time, this study reports BC concentrations and aerosol optical parameters near dense coal-fired power plants and open cast coal mining adjacent to the east IGP. In-situ measurements were carried out in Singrauli (located in south-east IGP) at a fixed site about 10 km from power plants and in transit measurements in close proximity to the plants, for few days in the month of January and March 2013. At the fixed site, BC concentration up to the 95 μgm^−3^ is observed with strong diurnal variations. BC concentration shows two maxima peaks during early morning and evening hours. High BC concentrations are observed in close proximity to the coal-fired TPPs (>200 μgm^−3^), compared to the outside domain of our study region. Co-located ground-based sunphotometer measurements of aerosol optical depth (AOD) show strong spatial variability at the fixed site, with AOD in the range 0.38–0.58, and the highest AOD in the range 0.7–0.95 near the TPPs in transit measurements (similar to the peak of BC concentrations). Additionally, the Angstrom exponent was found to be in the range 0.4–1.0 (maximum in the morning time) and highest in the proximity of TPPs (~1.0), suggesting abundance of fine particulates, whereas there was low Angstrom exponent over the surrounding coal mining areas. Low Angstrom exponent is characterized by dust from the unpaved roads and nearby coal mining areas. MODIS derived daily AOD shows a good match with the MICROTOPS AOD. The CALIPSO derived subtypes of the aerosol plot shows that the aerosols over Singrauli region are mainly dust, polluted dust, and elevated smoke. The preliminary study for few days provides information about the BC concentrations and aerosol optical properties from Singrauli (one of the NO_2_ hotspot locations in India). This preliminary study suggests that long-term continuous monitoring of BC is needed to understand the BC concentrations and aerosol optical properties for better quantification and the estimation of the emission to evaluate radiative forcing in the region.

## 1. Introduction

Incomplete combustion of fossil fuels, (e.g., diesel and coal), various types of biomass/biofuels in small-scale residential cooking and heating activities, and large forest fires/crops residue burning release huge amounts of black carbon (BC) aerosols [1,2,3,4]. Black carbon emissions are considered as particles, not a greenhouse gas, and they are the second or third largest climate warmer after carbon dioxide [5,6]. Its presence in the atmosphere causes poor visibility, a source of dense haze, fog and smog, that influence climate, affect human health and vegetation.

In recent years, the BC emission has attracted the interests of scientists due to its different anthropogenic sources and adverse effect on climate and environment [7]. Being in absorbing part of carbonaceous aerosols BC has different optical and radiative properties as compared to the other aerosols [8]. The atmospheric BC is directly responsible for the heating of the atmosphere by absorbing the short-wave solar radiation [9]. The uncertainties in the climate forcing from black carbon aerosol are largely due to inadequate study and measurements of cloud interaction with the co-emitted organic carbon [6]. The main removal mechanism of BC from the atmosphere is the wet deposition [10]. The chemically inert and fine size range particles are easily deposited by the rain. The average atmospheric lifetime of BC is about one week in the lower troposphere, depending on the ambient weather conditions, like wind speed, planetary boundary layer (PBL) height, humidity, etc., and also it depends on long-range transport [6,10]. The planetary boundary layer is the part of the atmosphere that is influenced directly by the earth’s surface and it responds to surface forcing with a timescale of about an hour or less [11]. Since most of the air pollutants are emitted, transported vertically, as well as horizontally into PBL, so it has a significant role in local air quality. Kalluri et al. [12] have shown that diurnal variations in BC concentration is strongly associated with daily changes in the local boundary layer in Anantapur.

The ground measurements of the atmospheric pollutant like BC at the regional levels (e.g., high polluted region Singrauli) are very much important. High concentrations of BC mass aerosols affect the rainfall pattern in the Asian monsoon system [13]. Recently, Li et al. [14] have carried out a comprehensive review on the aerosol and monsoon climate interaction over the Asian region and have explained that how the aerosols interact and can modify the amplitude, frequency, and intensity of the monsoon, with the help of observational and modeling data. Real-time monitoring of BC is useful in estimating the impact of BC on climate, environment, and human health. BC shows a certain pattern of diurnal as well as seasonal variations in different parts of India [15,16]. The coal power plant emissions are one of the main sources of dense haze, fog, and smog conditions in China and India. The black carbon concentration absorbs Sun radiations and impacts global climate as well as regional climate and monsoon [6,17]. Coal-fired power plants are densely located in the Indo-Gangetic Plain (IGP) and are considered as one of the sources of atmospheric pollution in the IGP [18,19,20,21,22,23]. The coal-fired thermal power plants are one of the major energy sources in India, the density of these power plants are very high in the northern and south-east parts of India, especially in the IGP. The BC emissions from TPPs are due to incomplete combustion processes. The fly ash from the power plants dispersed up to a radius of 10 km and affecting the air quality and human health.

The present paper reports the first preliminary ground-based in-situ measurement results of BC and aerosol optical properties from one of the highest pullulated regions with number of coal fired power plants. Due to many logistic issues, our measurements are limited to a few days. Our preliminary results show very high BC concentrations near the Thermal Power Plants (>200 μgm^−3^) and also corresponding a very high (~1.0) aerosol optical depth (AOD) measured using portable Aethalometer and handheld MICROTOPS sunphotometer. This study has much importance to the Indian scientific community, so that the Government and funding agencies consider the region and learn how people are living in such a polluted region. Due to severe pollution, only few measurements are carried out in the region with coal-fired power plants and coal mining. We also compared our results from the satellite-based (MODIS, CALIPSO) observations.

## 2. Material and Methods

### 2.1. Site Description and Surface Meteorological Conditions

**Figure 1:**
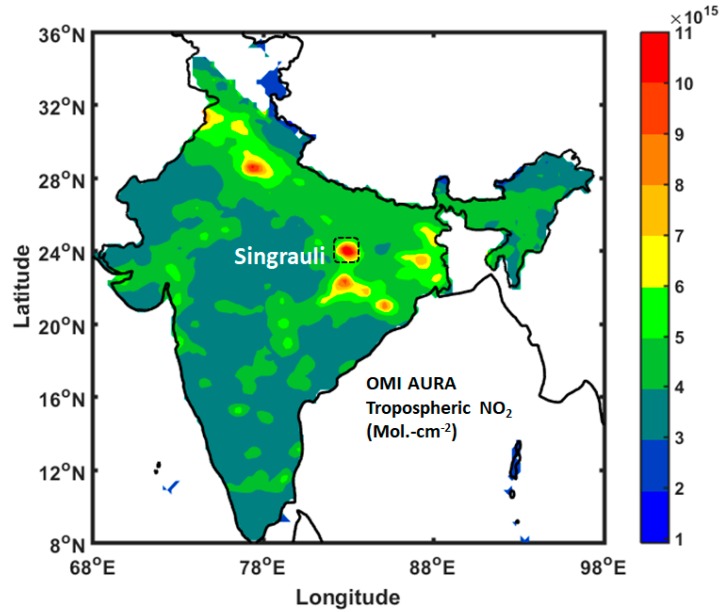
OMI derived average NO_2_ concentration over Indian region (68–98° E, 8–36° N) for 1 January–31 March 2013; black rectangle area represents the Singrauli and surrounding region.

OMI AURA satellite observation of NO_2_ concentrations [19,24] over India shows high concentrations at few locations, one of the locations correspond to the location of the seven coal-fired power plants (Singrauli). The NO_2_ and AOD show higher correlation based on satellite data [19,20,21,25]. The higher tropospheric NO_2_ concentrations over the Singrauli area, seen from the OMI AURA satellite (Figure 1) is considered for the in-situ BC and aerosol measurement. Singrauli (24.20° N, 82.66° E; 376 m above mean sea level), area lies in the eastern part of the state of Madhya Pradesh and the adjoining southern part of Sonebhadra district in the state of Uttar Pradesh is collectively known as Singrauli. The Singrauli area has emerged as India’s energy capital with seven coal-based power plants.

**Figure 2:**
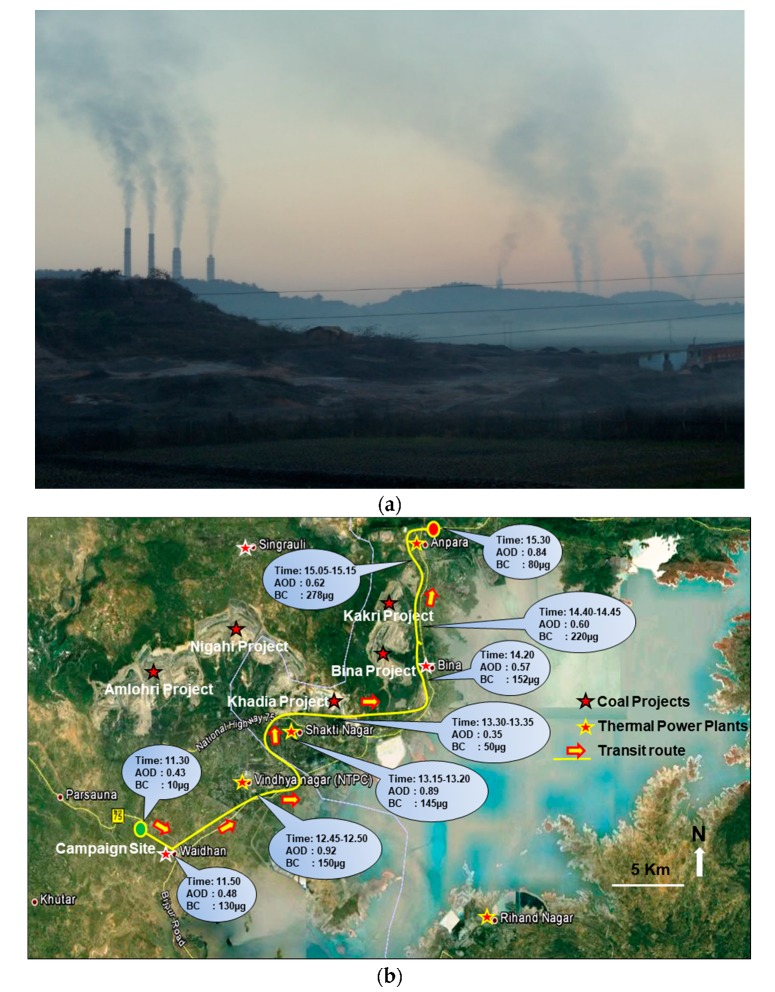
(**a**) The clear view of coal-fired power plants located in the Singrauli area (photo is taken by the main author—RPS); (**b**) The satellite picture showing the Singrauli region (black rectangle area in Figure 1), the fixed campaign site (Green filled spot), transit measurement route (Yellow line), power plants, coal mining areas, and GB Pant water reserve with corresponding time (h), AOD and BC values (µgm^−3^). (Picture Source: Google map).

Figure 2a shows the photo of the coal-fired power plants within a radius of about 20 km. The coals used by these power plants are from the coal reserves located in the proximity of power plants. Singrauli area generates around 35,000 MW of electrical power.

Major companies operating at Singrauli are: NTPC (National Thermal Power Corporation) (3 Power Plant with combined generation capacity of 9760 MW), Coal India Limited (through its subsidiary Northern Coalfields Limited annual production 80 Million MT), Reliance Power Limited (3960 MW), Essar Power Limited (1200 MW), DB Power Limited (1320 MW), Renusagar Power Plant (700 MW), Anpara Power Plants (Combined capacity of 3830 MW), Obra Thermal Power (1300 MW), Rihand Hydro Power (300 MW), and Hindalco Industries Limited (20k MT of Aluminum and 40k MT of Alumina per annum). Apart from these industries, there are many more small industries operational in the Singrauli area (http://www.singrauli.nic.in/). The main power plants, coal fields, and the GP Pant water reserve along the campaign site and routes are shown in Figure 2b.

**Figure 3:**
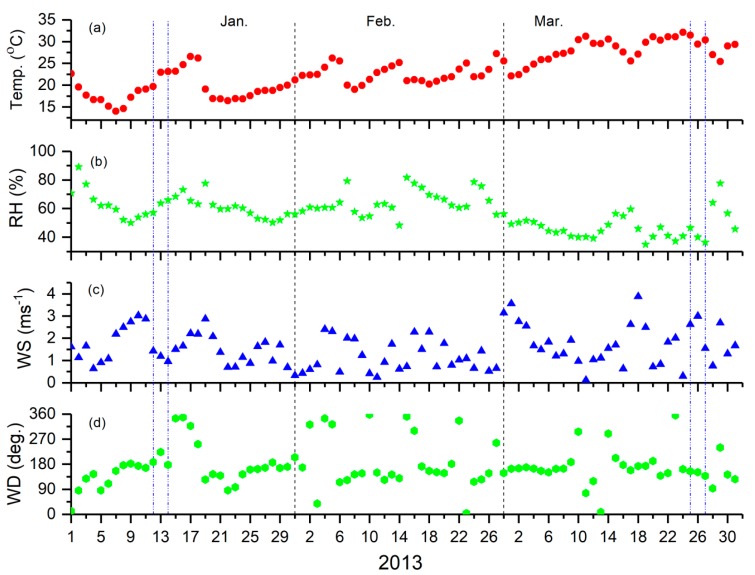
Day-to-day variability in the surface meteorological parameters (**a**) temperature (temp. °C), (**b**) relative humidity (%) (RH), (**c**) wind speed (ms^−1^) (WS), and (**d**) wind direction (deg.) (WD) at Singrauli during January–March 2013.

The surface meteorology plays an important role in the dispersion of aerosols into the atmosphere at any location. The ambient atmospheric condition over the Singrauli was studied during the study period using surface meteorological parameters, such as air temperature (temp.), relative humidity (RH), wind speed (WS), and wind direction (WD). These meteorological parameters were obtained from the ERA-Interim global analysis (from European Centre for Medium-Range Weather Forecasts or ECMWF) data (http://apps.ecmwf.int/datasets) with 0.25° spatial resolution [26]. The met data is used for understanding the distribution of near-surface temperature, RH, and WS and WD. Day-to-day variability in the above meteorological parameters are shown in the Figure 3a–d for January to March 2013. Values of different met parameters during measurements days are shown in Table 1.

During the measurement days (Figure 3a; shown between blue dash vertical lines), the temperature was found to vary in the range 19 and 31 °C. The lowest temperature over the Singrauli was observed on 12 January (19.70 °C) and highest on 25 March (31.44 °C). The relative humidity (Figure 3b) was generally found to be negatively correlated with the temperature in the month of March and was positively correlated in January and varies in the range 36 and 65% on measurement days. It was found to be highest on 14 January (65.92%) and lowest on the 27 March (36.42%). Surface winds (Figure 3c,d) show a mixed variation with the north-west direction in March and west to south-west in January. WS was found to be highest on 25 March (2.62 m/s) and lowest WS was observed on 14 January (0.95 m/s), with mostly from North-West and West direction, respectively.

The measurements were carried out in two modes (a) at a fixed location (near to Waidhan latitude 24.07° N, longitude 82.62° E) continuous measurement at a height of 2.43 m (eight feet). This location is about 10 km away south-west to TPPs and 7 km south to the coal mines. The measuring site is far from the road and also from the houses, mostly agricultural field (b) In transit measurement of BC and AOD along the road around power plants and coal mines (Figure 2b). We have started to drive from our fixed location (green spot shown in Figure 2b) after 11:45 h and followed the route shown in Figure 2b up to Anpara (red spot, south to north) till 15:30 h on 14 January 2013.

### 2.2. Instruments and Data

#### 2.2.1. Ground-Based

##### Aethalometer

We have used a portable aethalometer (Magee Scientific, Model AE51, wavelength 880 nm) for the black carbon measurements at fixed campaign site and along transit routes. This instrument records data automatically near real time, filter based measurement of BC mass concentration [27,28]. The aethalometer was set up in two positions (1) Fixed position at a height of about 2.43 m above the ground; (2) In a moving car with its outlet in the air at 1.52 m (~5) above the ground. The BC data were recorded with a sampling rate at 1 min (moving) and 5 min (fixed). The details about the instrument and the working methodology are well explained by Ferrero et al. [27] and Chakarabarty et al. [28]. The filter was changed after eight hours and also along different routes while making a set of measurements, a new filter was used. The flow of the aethalometer was kept 50 mL/min due to higher BC concentrations. The same model of aethalometer was used extensively by Praveen et al. [29] in India and the results were compared with the different models of aethalometer. We were limited with single wavelength aethalometer, however, initially, we did compare the results of the similar type of aethalometer and results were found to be same under similar conditions. The aethalometer was calibrated and tested before we made extensive measurements along the TPPs in Singrauli, India. Even though there may be some uncertainties in the BC measurements depending on the optical properties of BC and relative humidity condition, the BC measurement technique were well summarized by Kanaya et al. [30].

The measurements were carried out during 12–14 January 2013 and 25–27 March 2013 at a fixed location near Waidhan about 10 km away from the power plant and at different locations around power plants using aethalometer mounted on a car. Winter is the fog period in north India. We have selected the winter and pre-monsoon season, because we want to measure the locally generated BC, since during this period due to shallow boundary layer and low convection the dispersion of BC to long distances is restricted and we can have the actual measurements of locally generated BC.

##### MICROTOPS (MT) Sunphotometer

MT Sunphotometers have been extensively used for aerosol studies over IGP location and have been well calibrated for accuracy in their retrievals. The field of view of the MT is 2.5°, while the full width at half maximum bandwidth at each of the AOD channels is 2.4 ± 0.4 nm. Typical errors in AOD measurements from MT are in the range of 0.02–0.03 [31], and the aerosol retrievals were performed from the instantaneous solar flux measurements using the instrument’s internal calibration. Sunphotometer measurements were taken at two sites; from Fixed (away) and in-transit near the power plants at different locations. At each location, three sets of spectral scans were taken at each time under cloudless skies. The mobile measurements (after 11:30 to 15:30 h) at an important location (TPPs and coal mines) are shown with corresponding AOD values in Figure 2b. More details about MT and data retrieval can be found in Kaskaoutis et al. [31].

#### 2.2.2. Satellite-Based

##### MODIS

Gridded Atmospheric Product, collection 6.1 (061) MODIS Level 3, (1° × 1°) Terra MODIS AOD 550 nm values were used over the Singrauli region following the Combined dark target and deep blue AOD algorithm [32]. More details about retrieval and data product are available in Levy et al. [32]. Prasad and Singh [25] have shown a satisfactory agreement between MODIS and Kanpur AERONET AODs, since about 72% of the retrievals fall within the expected uncertainty of ±0.05 ± 0.15 × AOD over land [32]. MODIS AOD 550 nm data were obtained via the Giovanni Online Visualization and Analysis system (http://disc.sci.gsfc.nasa.gov/giovanni).

##### OMI

In addition to MODIS, Aura OMI daily NO_2_ total column and tropospheric column were used for identifying the hotspot of emission over India. Spatial resolution for OMI is 0.25° × 0.25° grid. The OMI algorithms for data retrieval are described by Irie et al. [33]. The comparison of OMI product with surface NO_2_ show satisfactory agreement, suggesting a bias of ±20% [33].

##### CALIPSO

The Cloud-Aerosol Lidar and Infrared Pathfinder Satellite Observation (CALIPSO) flies at an altitude of 705 km with a 98° inclination orbit providing new insight in atmospheric monitoring by observing the vertical profiles of aerosols and cloud [34]. The CALIOP Level 1 (version 4.10) aerosol subtypes for the identification of pollutant type and vertical distribution (http://www-calipso.larc.nasa.gov/products/) was used in the present study for a typical day over Singrauli region during the campaign in March 2013. The CALIPSO calibration and uncertainty, as well as the CALIPSO data products, are discussed in detail by Roger et al. [35].

## 3. Results and Discussion

### 3.1. Temporal Variation of BC_880 nm_ and AOD

#### 3.1.1. Diurnal Variation of BC

**Figure 4:**
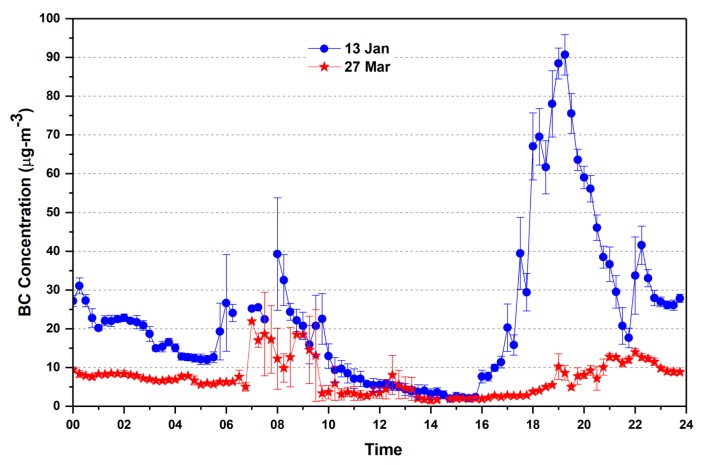
Diurnal variation of black carbon (BC) concentration at fixed campaign site on 13 January and 27 March 2013 (averaged for 15-min with standard deviation).

The 24-h continuous measurement of BC concentration at the fixed site is shown in Figure 4 for the two different campaigns during winter (13 January) and pre-monsoon season (27 March). BC was measured for every five-minute interval and averaged for fifteen minutes. In January BC concentrations at the campaign site shows two peaks in the morning and evening hours, up to 40 μgm^−3^ in the morning hours (6:00–10:00 h) and up to 90 μgm^−3^ in the evening hours (17:00–22:00 h). A gradual decrease with minimum BC mass concentration up to the 2.56 μgm^−3^ during the afternoon hours, followed by an increasing trend after the local sunset was seen. Similar variation was recorded for the March also but the morning and evening peak was less prominent, due to the change in boundary layer height and less calm environment (as shown in Figure 3 and Table 1), which allows for diffusing the pollutants for long distances and heights. The diurnal mean BC mass concentration found around 23.36 μgm^−3^ in the month of January and 7.3 μgm^−3^ in the month of March 2013. The average mass concentration is higher when compared to the previous studies for the almost similar environment (in the vicinity of the coal mine and TPPs) in Dhanbad (~10 μgm^−3^) by Singh et al. [4,36].

#### 3.1.2. Night Time Variation of BC

**Figure 5:**
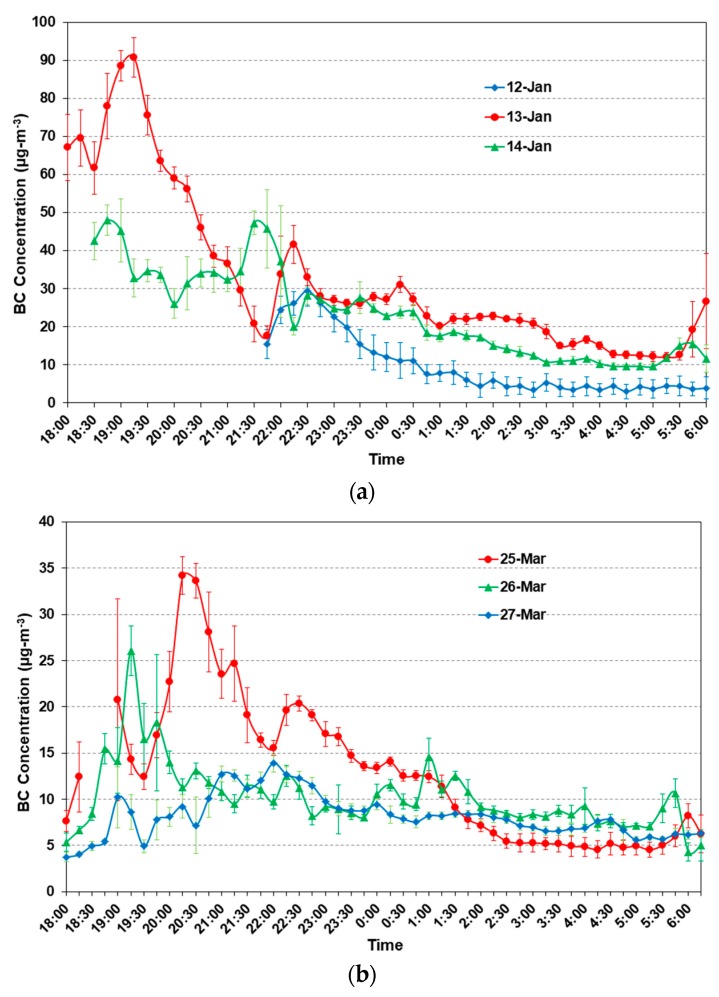
(**a**) Night time BC profile with standard deviation (15 min average), observed at fixed campaign site for the days 13, 14 and 15 January 2013; (**b**) Same as (**a**), but for 25, 26 and 27 March 2013.

The night time variation of BC is shown in Figure 5 for the month of January and March campaign. The evening peak pattern was more pronounced during the winter season as compared to the pre-monsoon season due to the weaker dispersion that is associated with slower wind speed (WS) and cooler temperature (Table 1) and enhanced biomass burning [37]. Night time concentrations of the BC particles were higher when compared to those during day time concentrations in both the cases. Large diurnal variability in BC has been linked with enhanced emissions and meteorological conditions, especially lower boundary layer heights, WS, and Temperature. In earlier studies [38,39] the winter-time peak (90 μgm^−3^) was much higher (~2 times) as compared to the pre-monsoon season peaks (35 μgm^−3^). The late-night BC concentration shows low values due to the decrease of vehicles on the road, close of industries, and night time cooking, and in the morning BC peaks are observed due to vehicles on the roads, coal mining activities, cooking, and operation of small industries.

During pre-monsoon, BC concentration peak in the evening shifted towards the late evening hours due to the shift of fall in temperature and the reduction in boundary layer height which is associated with seasonal and meteorological condition at the site, but an elevated peak is observed on 26 March between 18:30 and 20:30 h, which may be associated with the burning of woods and biomass. This may be attributed to the pre Holi burning (Holika) when the massive wood and carbonaceous materials are burned in India for celebrating the Holi festival [40].

#### 3.1.3. BC and AOD Comparison

**Figure 6:**
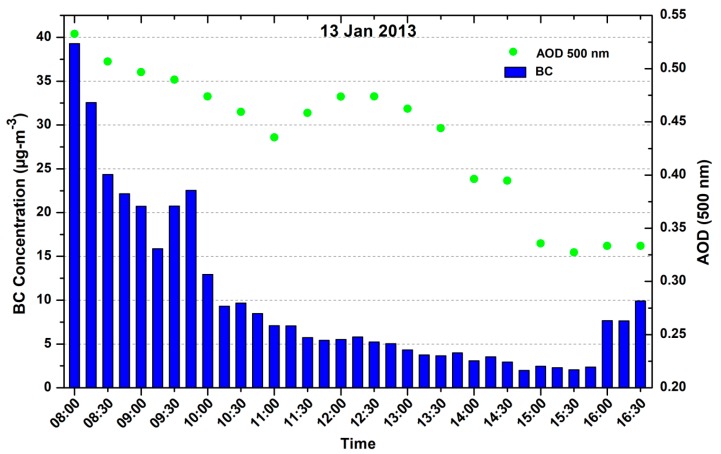
Day time (h) aerosol optical depth (AOD) (500 nm) and BC variation over the fixed measurement site in Singrauli.

Since BC is also an aerosol and can affect the optical properties of the atmosphere, so to check any association of BC with AOD, we have shown diurnal variation of BC and AOD for 13 January 2013 in Figure 6. Measurement for AOD at 500 nm was taken at 30 min interval. High AOD (>0.5) was observed in the morning time and afterward gradually decreased and increased at the noontime and then decreased, this could be due to diffusion process that is associated with the increasing temperature as the day progresses. Similarly, the BC concentration was high in morning hours and gradually decreased up to evening (with little increase in noon) and again started to increase after sunset due to the decrease in temperature and boundary layer (BL) height, which restrict the dispersion of the BC. The role of the BL dynamics in causing the observed diurnal variations has been well-documented over Indian region [41,42]. The diurnal pattern is most pronounced during winter season.

#### 3.1.4. MODIS and MICROTOPS AOD Comparison

We have compared AOD measured from MICROTOP with the MODIS Terra AOD for 13–14 January. The evaluation of AOD from MICROTOPS is shown in Section 2.2.1. The average ground-based AOD was 0.43 and for MODIS the average value was 0.47 over the Singrauli region showing reasonably good match for the region.

### 3.2. First In-Situ Measurement of BC and AOD in TPPs Vicinity

**Figure 7:**
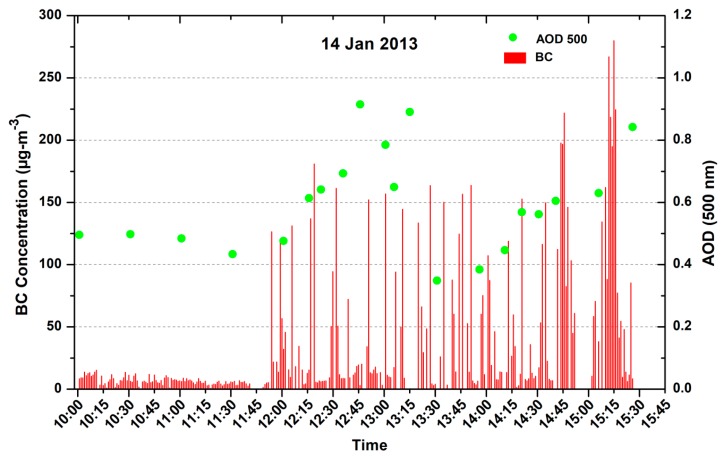
Measurement of AOD (500 nm) and BC at fixed site (10:00–11:45 h) and in transit near the thermal power plants (11:45–15:30 h).

Figure 7 shows the in-situ transit measurement of BC and AOD in close proximity of the TPPs. The AOD (500 nm) variations from MICROTOPS along BC values are shown on 14 January from 10:00 to 15:30 h. The low values of AOD from 10:00 to 11:30 h are from the fixed site location ~10 km away from the TPPs (green spot on Figure 2b). The AOD values after 11:30 to 15:30 are from the in-situ transit measurement of AOD near the TPPs (yellow route in Figure 2b). Similar variation for the BC is from 10:00 to 11:45 h at the fixed site and after 11.45 h transit measurements in the proximity of the TPPs in a moving car. The high value of BC and AOD at 12:40, 13:20, 14:20, 14:40, and 15:15 h are associated with the measurement near the Vindhya Nagar, Shakti Nagar, Bina, and Kakri coal project and before Anpara power plants. The lowest values of AOD and BC at 13:30 are associated with the forest region between the Shakti Nagar power plant and the Bina coal field mining area.

The BC concentration near the TPPs are observed to be much higher (>200 μgm^−3^) in comparison with the places that are away from the sources. The peak BC concentrations reached as high as 278 μgm^−3^ near the Anpara TPP. The effect of the emission was also seen in the AOD measurement near (~1.0) and away (0.5) the TPPs. Higher AOD is found at the TPPs and surrounding area and low values were observed far away in all of the wavelengths.

**Figure 8:**
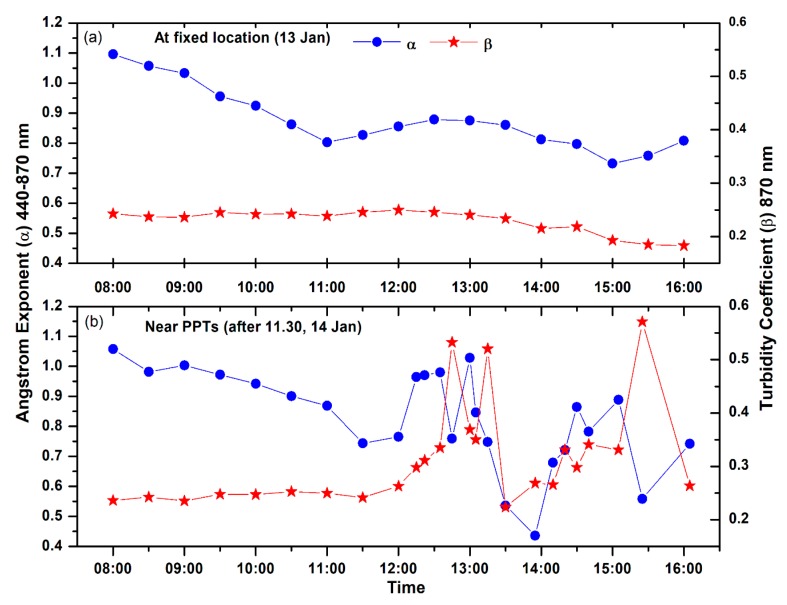
(**a**) Variation of Angstrom exponent (α) and Turbidity coefficient (β) at the fixed campaign site. (**b**) Same as Figure a, but for the in-situ measurement (after 11:30) near the thermal power plants and coal mining areas.

We started our transit measurement from fixed campaign site (green spot in Figure 2b) after 11:45 h, earlier the BC concentration was about the 20 μgm^−3^. The BC concentration shows a low value below the 10 μgm^−3^ as we entered in the shopping area. BC concentrations increased near the Vindhya Nagar power plant, the maximum BC concentrations shoot up to 150 μgm^−3^. The average value of BC was ~100 μgm^−3^ during the transit measurement period. After Vindhya Nagar, the BC values were about 145 μgm^−3^ near the Shakti Nagar power plant. Further, the BC concentration decreased close to forest area between Shakti Nagar and Bina coal project at 13:30 h. Low AOD was also observed, but the reduction in the alpha value (Figure 8b) shows the dominance of coarse particles that are associated with the coal dust. Average BC concentration varies in the range 60–90 μgm^−3^ along the Bina and Kakri coal mining areas. When we were approaching Anpara (Figure 2b), the BC concentrations abruptly enhanced up to 220–250 μgm^−3^ and reached a maximum value 278 μgm^−3^ close to the Anpara power plant where the transportation of coal was carried out using open trucks by the road for the Anpara power plant (14:45 and 15:15 h). When we reached the Anpara power plant the BC value was ~50 μgm^−3^. The low concentration of BC was observed in the proximity of the Anpara power plant, due to the height of the chimneys (about 200 m) and the westerly wind, however high AOD value was observed in the proximity of power plant. The height of chimneys of the plants are around 200 m so the plume of smoke is transported by air towards the places that are little away from the plant (~3 km), depending upon the winds so low BC concentration was observed. The AOD observed from MICROTOPS was very high (~1.0) since it measures the vertical distribution of AOD at the power plants and less around the plants due to the rapid diffusion of the plume. The present results clearly show that the coal-fired power plants are the main sources of high BC concentrations and high aerosol optical depth, which is somehow ignored from the black carbon emission inventory in India. Similar high BC concentration was also reported for coalfield and TPPs area of the Dhanbad region [4].

### 3.3. Angstrom Exponent and Turbidity Coefficient Variation at the Fixed Site and in Transit Near the TPPs

Angstrom suggested an empirical formula for the attenuation of scattering and absorption by aerosols. According to his formula, the AOD, τ (λ), is related to wavelength (λ in μm) through Angstrom’s equation:

τ (λ) = βλ^−α^, (1)

where α and β are known as Angstrom exponent and Angstrom turbidity coefficient. The Angstrom exponent, α, is related to the size distribution of the aerosol particles. Large values of α indicate a relatively high ratio of small particles to large particles. It varies in the range of 0–4, approaching 4, when the aerosol particles are very small (i.e., the order of air molecules) and zero for very large particles. Mid-range values of α (α > 2) are typically observed for particles in the accumulation mode [43] and lower values (near to 0) have been observed for Saharan and Thar dust episodes and coarse mode particles [44,45].

The Angstrom turbidity coefficient (β) represents the amount of aerosols that are present in the atmosphere in the vertical direction, and generally, its value varies from 0.0 to 0.5. The value of β < 0.1 indicates a clean atmosphere, while β > 0.1 shows a turbid atmosphere. In fact, according to Equation (1), β is the AOD at λ = 1 μm. Therefore, AOD and β usually have a similar variation. After converting the Angstrom Equation (1) to a logarithmic format, it yields:

ln τ(λ) = ln β − αln λ.(2)

There are many techniques to determine the values of β and α. The most accurate method is to measure the AOD at two wavelengths. Here, we have selected wavelengths 440 and 870 nm.

The Angstrom exponent and turbidity coefficient are shown in Figure 8a (at fixed site) and 8b (In-situ transit measurement after 11:30 h). The Angstrom exponents were computed while using the spectral AODs at all the five wavelengths (380, 440, 500, 675, 870 nm) from MICROTOP. The mean wavelength exponent (α_440–870 nm_), obtained was around 0.85 ± 0.14 (Figure 8). The mean alpha for the fixed site and in transit measurement was, respectively, about 0.89 ± 0.10 and 0.78 ± 0.17. The alpha varies in the range 0.75 to 1.10 (Figure 8a), the highest value of alpha in the morning hour and decreases around 11:00 and afterward increased up to 0.90 at 13.00 h and again decreased till 16:30 h. On the next day, alpha varies from 1.10 in the morning to 0.75 around noon time. High values of alpha indicate the presence of fine mode particles (Figure 8a). At different locations, along the road from Waidhan, alpha varies from 0.45 to 1.02 (Figure 8b), high alpha near power plants showing a dominance of fine particles from the plume of power plants and the low values of alpha near the coal mining areas shows the presence of coarse particles from the coal dust.

The mean turbidity parameter (β) represents the total aerosol loading in the atmosphere found 0.28 ± 0.09 at our fixed site. The mean β values at the fixed site (Figure 8a) and along the road in the vicinity of TPPs (Figure 8b), respectively, vary in the range 0.23 ± 0.02 and 0.34 ± 0.10. High beta values near the power plants show high vertical loading of aerosols as compared to other places. Low values of beta between Shakti Nagar and Bina coalfield areas show low vertical loading due to a small patch of forests on both sides of the road (Figure 2b and Figure 8b). A contrast difference in beta values is observed at fixed campaign site (Waidhan) in the range 0.17–0.26 and within power plants area in the range 0.23–0.58. High peaks over the power plants, three peaks corresponding to three TPPs (Figure 8b) show high vertical aerosol loading from these power plants. The alpha and beta values are anti-correlated near the thermal power plants can be attributed to high aerosol loading and relatively coarser particles due to mining activity and unpaved road dust.

### 3.4. Identification of Pollutants by CALIPSO Profile

**Figure 9:**
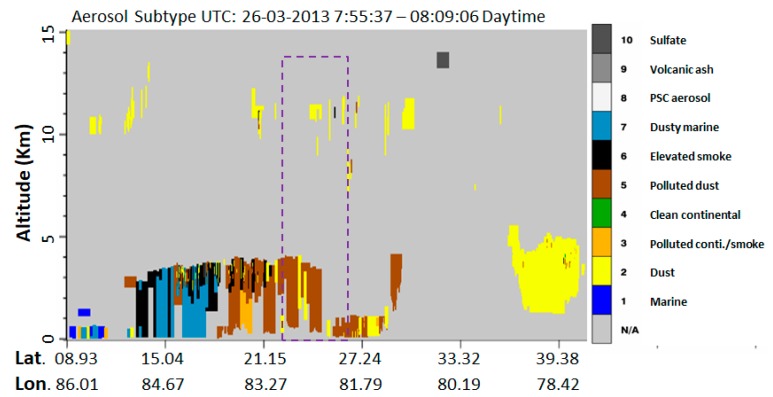
Cloud-Aerosol Lidar and Infrared Pathfinder Satellite Observation (CALIPSO) satellite aerosol types, and vertical distribution recorded on 26 March 2013.

The CALIPSO satellite overpass (space-time cross-sections of aerosol vertical feature mask (VFM)) available for 26 March 2013 (Pre Holi day) is shown in Figure 9. The area with the violet rectangle represents the observation site at Singrauli and its surroundings. The vertical distribution of aerosols from the CALIPSO satellite clearly shows a presence of dust, polluted dust, and elevated smoke within Singrauli region, where a number of power plants emit smoke, soot particles, and coal dust from the mining activities. The coal power plants can be identify as a different pollutant source in comparing to the surface emission sources and the pollutants can penetrate deep in the atmosphere due to the stake height of the power plants. Once these aerosols are reached up to the 4–5 km in vertical height, they can stay there longer period. These different types of aerosols can interact with the convection and cloud and can change the microphysics and their optical and radiative properties.

## 4. Conclusions

This study presents BC concentrations and aerosol parameters from Singrauli, a site surrounded by seven coal-fired power plants and coal mining areas located in the southern edge of IGP. OMI satellite clearly shows the study areas as a hotspot region for the NO_2_ emission. The main findings are summarized, as follows.

1. BC concentration shows a well-defined diurnal variation with two peaks, one in the morning around 06:00–10:00 h and the other in the evening between 17:00 and 22:00 h. In addition, there is a distinct decrease in the early morning hours. Reduction in PBL and wind speed is the causative factors for the accumulation of aerosol particles in the lower layer. Moreover, early morning hours may be characterized by high relative humidity conditions, and hence the accumulated aerosol particles coagulate and become heavier, thereby resulting in a decrease in concentration. The mean concentrations of BC during January and March months at the fixed location are about 23.36 and 7.3 μgm^−3^, respectively.
2. The BC concentration near the power plants was recorded much higher (>200 μgm^−3^) compared to the observed concentrations at the fixed site (~10 km away from the power plants). The average AOD at the fixed site was 0.43, which shows a reasonably close match with the MODIS derived AOD (0.47). High AOD value of ~1.0 was observed in transit measurement near the power plants for 500 nm wavelength at some places.
3. Alpha and Beta variation at the fixed site and in transit along the road shows the dominance of fine particles and high loading of aerosols, while lower values of both the parameters show less polluted and the presence of coarse particles near the forest areas.
4. The CALIPSO aerosol sub-types observation clearly shows a dominance of dust particles, polluted dust and smoke vertically over the Singrauli region.
5. Depending on weather parameters in the winter months the pollutants diffusion is less and they get suspended near to the surface and that enhances the BC concentration, while in pre-monsoon month (March), the emitted pollutant disperse more in height and distance and thus a low concentration of BC was observed.
6. Our preliminary results are limited to only a few days due to various constraints (logistics issue), which clearly show high BC concentrations and high AOD in the Singrauli area. Detailed measurements for a longer period are required to understand the day to day variability of BC and AOD with meteorological parameters and to study the effect of burning of coal in power plants on the BC concentrations and AOD. Long-term observations will be of great help in the model estimation of BC and AOD from coal-fired power plant areas.
7. The measurements of BC (within the uncertainties limit) could be helpful in improving the accurate emission inputs in climate models. High atmospheric pollution and poor air quality are a threat to human health in the area.

## Figures and Tables

**Table 1:** Values of the surface meteorological parameters temp., RH, WS, and WD at Singrauli during measurement days of January and March 2013.

Day/Met. Parameters	Temp (°C)	RH (%)	WS (ms^−1^)	WD (deg.)
12 January	19.70	57.14	1.42	187.33
13 January	22.95	63.67	1.20	223.59
14 January	23.09	65.92	0.95	177.80
25 March	31.44	46.67	2.62	154.37
26 March	29.40	40.00	2.99	151.18
27 March	30.38	36.42	1.54	137.93
